# Development of the Food Label Information Program: A Comprehensive Canadian Branded Food Composition Database

**DOI:** 10.3389/fnut.2021.825050

**Published:** 2022-02-03

**Authors:** Mavra Ahmed, Alyssa Schermel, Jennifer Lee, Madyson Weippert, Beatriz Franco-Arellano, Mary L'Abbé

**Affiliations:** ^1^Department of Nutritional Sciences, Temerty Faculty School of Medicine, University of Toronto, Toronto, ON, Canada; ^2^Faculty of Health Sciences, Ontario Tech University, Oshawa, ON, Canada

**Keywords:** branded food database, food composition, packaged foods, artificial intelligence, Optical Character Recognition, nutrition facts table, web-scraping

## Abstract

**Objectives:**

Traditional methods for creating food composition databases struggle to cope with the large number of products and the rapid pace of turnover in the food supply. This paper introduces Food Label Information Program (FLIP), a big data approach to the evaluation of the Canadian food supply and presents the latest methods used in the development of this database.

**Methods:**

The Food Label Information Program (FLIP) is a database of Canadian food and beverage package labels by brand name. The latest iteration of the FLIP, FLIP 2020, was developed using website “scraping” to collect food labeling information (e.g., nutritional composition, price, product images, ingredients, brand, etc.) on all foods and beverages available on seven major Canadian e-grocery retailer websites between May 2020 and February 2021.

**Results:**

The University of Toronto's Food Label Information Program (FLIP) 2020 was developed in three phases: Phase 1, database development and enhancements; Phase 2, data capture and management of food products and nutrition information; Phase 3, data processing and food categorizing. A total of 74,445 products available on websites of seven retailers and 2 location-specific duplicate retailers were collected for FLIP 2020. Of 57,006 food and beverage products available on seven retailers, nutritional composition data were available for about 60% of the products and ingredients were available for about 45%. Data for energy, protein, carbohydrate, fat, sugar, sodium and saturated fat were present for 54–65% of the products, while fiber information was available for 37%. Food products were classified under multiple categorization systems, including Health Canada's Table of Reference Amounts, Health Canada's sodium categories for guiding benchmark sodium levels, sugar-focused categories and categories specific to various global nutrient profiling models.

**Conclusions:**

FLIP is a powerful tool for evaluating and monitoring the Canadian food supply environment. The comprehensive sampling and granularity of collection provides power for revealing analyses of the relationship between nutritional quality and marketing of branded foods, timely observation of product reformulation and other changes to the Canadian food supply.

## Introduction

The study of nutritional epidemiology relies on understanding the association between nutrient consumption and health outcomes and usually involves monitoring the nutritional quality of food consumed by a population (1). Thus, such studies rely on the assessments of dietary intakes based on the collection of nutrition information from food composition tables or databases (1). Packaged foods and beverages represent a major segment of the food supply, providing approximately two-thirds of energy intake (2, 3). Despite the dominant role of these packaged foods and beverages in the diets of populations, existing food composition databases are limited in their ability to capture accurate nutrient content information for specific products due to the complex and dynamic nature of the national food supplies (4–6).

National food composition databases are expensive to develop, construct and maintain (7, 8). The packaged food and beverage sector includes a wide array of products and is characterized by continuous changes and turnover due to introduction of new products, reformulation or discontinuation of others (8, 9). Most national food composition databases include aggregate nutrition information for only a limited number of generic food items. For example, to assess dietary intakes of Canadians, researchers rely on the Canadian Nutrient File (10) to estimate the dietary intake component of dietary data, including 24-h recalls, food frequency questionnaires (11, 12), and other national nutrition surveys, [e.g., Canadian Community Health Survey (CCHS) (13)]. The CNF database is the standard reference food composition database developed and maintained by the Government of Canada, and is used by a number of Government of Canada agencies including Statistics Canada, Health Canada, Agriculture and Agri-Food Canada, and the Canadian Food Inspection Agency (10), industry and researchers. Nutrient information for all food and beverages reported by CCHS participants comes from the CNF, which is composed of nutrient profiles for about 6,000 products that are primarily generic representative composites, where more than half are recipe-based foods/beverages based on common preparation methods, rather than individual food/beverage items (14). However, using the CNF to analyze changes in the food supply or Canadian population dietary intakes poses several challenges due to its lack of scheduled comprehensive and systematic updating, the use of some non-Canadian food composition data, and aggregated data for packaged foods.

The packaged food and beverage industry is also characterized by fast-moving continuous turnover as new products are introduced and/or reformulated, some to replace less-favored or discontinued products (5, 6, 15–18). These continuous changes require food composition databases to be updated frequently; however, lack of resources limits the updating of the nutritional composition of all foods in many food databases, especially for foods found in the generic CNF. Globally, there have been various attempts to collate such large-scale up-to-date nutritional data on a comprehensive set of foods: for example, by crowdsourcing food label data using mobile phones [e.g., FoodSwitch in Australia (19)] or web applications [e.g., Open Food Facts in the United States (20)]; collecting data through contact with food manufacturers/industry (e.g., the USDA Global Branded Food Products Database) (21–23); or periodic audits of the foods on the market (e.g., FoodDB extracted weekly nutrition information on products using web-scraping in the United Kingdom) (24). However, there are limited developments of food composition databases that achieve comprehensive coverage of the Canadian supply system with brand-specificity and regularly up-to-date, extensive nutrition information of food products.

To address these research gaps, we aimed to develop a product- and brand-specific comprehensive database containing nutrition information for a diverse array of packaged foods and beverages in the Canadian food supply. Such a database allows for identifying important levers for promoting healthy diets, prioritizing nutritional interventions for public health policy, evaluating the impact of population-level policies such as Sodium Reformulation (25) or Trans Fat Ban (26) and effects of future policy interventions such as front-of-pack labeling (FOPL) or Marketing to Kids (M2K). It can also be used in national nutrition surveys to access the nutritional quality of diets of Canadians and assess the changes in nutritional composition of the food supply in response to policy or other changes such as the COVID-19 pandemic.

Previously, we have developed three versions of FLIP datasets in 2010, 2013, and 2017, described in detail elsewhere (26–28). However, in these previous versions, data were collected in stores using a smartphone application in 2013 and 2017, while in 2010, data from the products (e.g., nutrition information) were manually entered into the database/website. Given the common usage of big data techniques in collecting, storing, processing and analyzing data, now applied in many fields across non-profit, scientific, business and public sectors, this paper introduces FLIP 2020, an Artificial Intelligence (AI)-enhanced/powered Optical Character Recognition (OCR) (AI-enhanced OCR) approach to the collection and evaluation of the Canadian packaged food and beverage supply and presents the methods used in the development of this database.

## Methods

**Figure 1:**
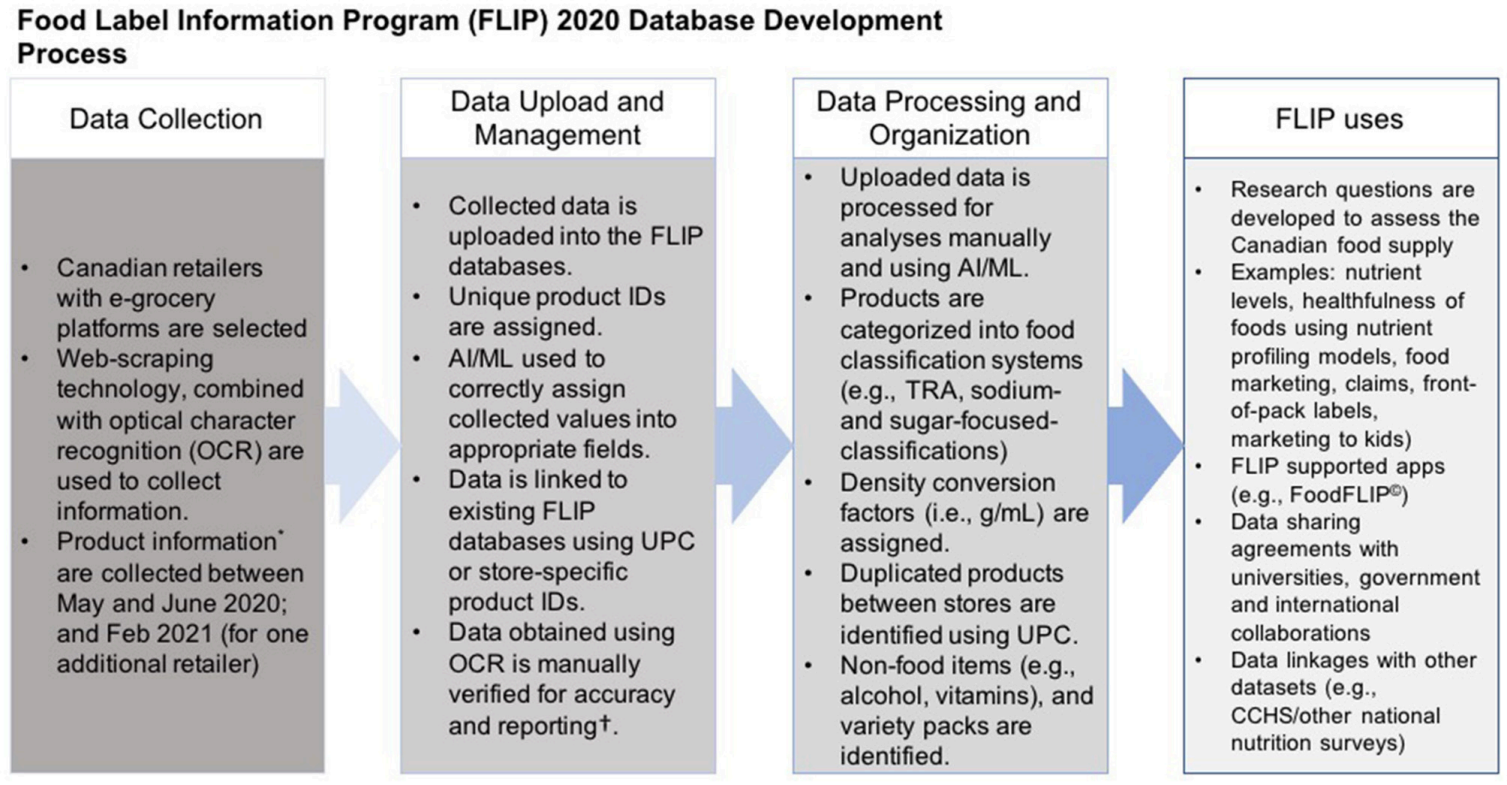
Phases of development of Food Label Information Program (FLIP) 2020. FLIP 2010, 2013, and 2017 are previous versions of FLIP.

The University of Toronto's Food Label Information Program (FLIP) 2020 was developed in three phases: Phase 1, database development and enhancements; Phase 2, data capture and management of food products and nutrition information (i.e., Nutrition Facts table (NFt); Phase 3, data processing and food categorization (Figure 1).

### Overview of Food Label Information Program Database

The FLIP is a database of Canadian pre-packaged food and beverage package labels by brand name that was updated every 3–4 years at the University of Toronto (UofT), Toronto, ON, Canada. The purpose of the FLIP is to provide comprehensive food product nutrition information to allow for assessment and monitoring over time. To date, three previous versions of the FLIP datasets have been completed in 2010, 2013, and 2017 and are described in detail elsewhere (26–28). Briefly, data for FLIP 2010, FLIP 2013 and FLIP 2017 were collected in person in stores and stored on the FLIP website, a database (website) to collect, process, store and manage the data. FLIP 2010/11 (*n* = 10,487) (27) was collected in the Greater Toronto Area (GTA) and Calgary between March 2010 and April 2011; FLIP 2013 (*n* = 15,342) (28) data were collected in the GTA, Ottawa and Calgary during May to September 2013; and FLIP 2017 (*n* = 17,671) (26) data were collected during July to September 2017 in the GTA. These collections represented 56% (27), 75% (28), and 68% (29) of grocery retail sales in Canada, respectively for 2010, 2013, and 2017. All nationally available and private-label brands, but excluding seasonal products, were collected from major retailers (Loblaws, Metro, Sobeys and Safeway in 2010 and 2013; Loblaws, Metro and Sobeys in 2017). While inclusion criteria for foods and beverages changed little between each FLIP version, data were collected using a smartphone application in 2013 and 2017, while in 2010, data from the products (e.g., nutrition information) were manually entered into the database/website (Table 1).

**Table 1:** History of the Food Label Information Program (FLIP) Database collections and updates.

**FLIP database**	**Collection period**	***n*** ^*^	**Number of stores**	**Collected product variables/information**	**Collection method**
2010–11	March 2010–April 2011	10,487	4	Name, Brand, Company, Container size, NFt, UPC, Marketing information^‡^	• Food packages purchased for data collection. • Variables of interest were manually entered in Microsoft Excel. • Uploaded to FLIP cloud database following the 2013 collection.
2013	May–Sept	15,342	4	Name, Brand, Company, Container size, NFt, UPC, Marketing information^‡^ Ingredients List, Photos of all sides of packages	• iPhone app development for digital collection of food package images in stores. • Database software development using Cloud storage. • OCR software development to automate NFt and ingredients list data entry. • Excel report generation capabilities added.
2017	July–Sept	19,267	3	Name, Brand, Company, Container size, NFt, UPC, Marketing information^‡^, Ingredients List, Price (regular & sale), Photos of all sides of packages	• Upgraded technology capabilities, including ability to update databases using Excel. • Automated linking & matching products between databases using UPC or store-specific product codes • Development of algorithms for food categorization and nutrient profiling
2020–21	May 2020–Feb 2021	74,445	9^†^	Name, Brand, Container size, NFt, UPC, Ingredients List, Price (regular & sale), Photo of front of package (if available)	• Web scraping to collect all product information • AI-enhanced OCR technology to collect all product information. • Automated linking & matching UPCs / store-specific codes between FLIP 2020 and FLIP 2017, and between stores within FLIP 2020

* *Sample sizes in the FLIP 2010–11, 2013 reflect unique products, while the sample size in FLIP 2017 also includes multiple package sizes and FLIP 2020 includes multiple package sizes and duplicates across stores*.

† *Data was collected from seven retailers plus two location-specific stores*.

‡ *Marketing information included nutrient content claims, health claims, front-of-pack labeling, and children's marketing. Tabs and options can be and have been expanded over time, depending on research needs*.

*FLIP, Food Label Information Program; NFt, Nutrition Facts table; OCR, optical character recognition; UPC, universal product code*.

The FLIP website enables users to generate data outputs and reports in Microsoft Excel for further analyses. The FLIP website contains a user tutorial, user guides, and a dashboard with the FLIP version number and details on the latest updates. The information captured on each product is described in Table 2.

**Table 2:** Information collected, managed and processed on the FLIP website.

**FLIP website tabs**	**Description of information**
Description	Product ID, company, brand, name, preparation required, variety pack, TRA categories, ingredients, sampling date, store code, container size and price
Barcode/UPC	Barcode/UPC, sample date, store code and linkages
Nutrition facts	Collected data: Serving size, weight/volume, nutrient contents as identified on the package Nutrition Facts table (amount and %DV) (Kcal, Fat, saturated fat, trans fat, cholesterol, sodium, potassium, carbohydrates, fiber, sugar, free sugar, protein, vitamin A, vitamin C, calcium, iron) Calculated data: weight/volume conversions (g/mL and mL/g e.g., density), as prepared nutrition information captured in 2013, 2017, nutrition information per 100 g
Marketing	Children's marketing, nutrient content claims, other claims, disease risk reduction claims, front of pack symbols, structure/function claims^*^
Nutrient profiling	Nutrient profiling and other information used in calculating nutrient profiling scores (e.g., FSANZ, Ofcom, UK TLL, Health Canada Surveillance Tool, FoodFlip (FoodFlip app related nutrient profiling models), added sugar, free sugar, PHO, sweeteners, NOVA Processing, added fats, whole grains^*^
Sodium [Categories]	Sodium-focused categories
Sugar [Categories]	Sugar-focused categories
Photos	Images of the product including front, back and sides, NFt, ingredient declaration (~8 photos per product as available)
Matches	Matched products with previous versions of FLIP
Log	User-inputted comments on validation and updates to the product

* *Tabs and options can be and have been expanded over time, depending on research needs*.

*FLIP, Food Label Information Program; FSANZ, Food Standards Australia New Zealand; NFt, Nutrition Facts table; TLL, Traffic Light Label; TRA, Table of Reference Amounts; UPC, Universal Product Code*.

#### Data Security

The FLIP is hosted on a cloud-based infrastructure located in Virginia, USA and Quebec, Canada. Raw data for each product page with date and time of data collection is stored separately for audit and data verification purposes, and to provide a mechanism for re-extracting data in the event that data was previously extracted incorrectly, or additional data points are required. At present, the FLIP website is available to the L'Abbé Lab nutritional sciences researchers at the University of Toronto, as well as national and international university and government researchers with whom the University of Toronto has set up data sharing agreements.

### Phase 1: Food Label Information Program Database Development and Enhancements

#### FLIP 2020 Data Collection

The latest phase, FLIP 2020, is described in this manuscript. The FLIP 2020 contains nutrition information for 74,445 product listings, representing 48,829 unique universal product codes (UPC). Food information from the leading grocery retailers in Canada with online information were acquired from their respective websites and digitalized to enhance ease and efficiency of collection and analysis. Food composition database software (University of Toronto, Toronto, ON, Canada) (web and mobile) was developed for FLIP 2020, resulting in a shorter and more efficient food collection and data processing approach (Table 1).

Data was acquired from the websites of seven Canadian retailers (Costco^®^, Costco Wholesale Canada Ltd., Nepean, ON, Canada; Grocery Gateway by Longo's, Longo's Brothers Fruit Markets Inc., Empire Company Ltd., Stellarton, NS, Canada; Loblaws^®^, Loblaws Companies Ltd., Brampton, ON, Canada; Metro, Metro Inc., Montreal, QB, Canada; No Frills^®^, Loblaws Companies Ltd., Brampton, ON, Canada; Voilà by Sobeys, Empire Company Ltd., Stellarton, NS, Canada; and Walmart, Walmart Canada Corp., Mississauga, ON, Canada), representing over 80% of the grocery retail market share (30, 31). Data were collected between May and June 2020, and in February 2021 (the latter for Voilá due to the lack of e-commerce availability during the initial scraping period). Two additional websites of two retailers (Loblaws and No Frills), located in a populated metropolitan Toronto area, were selected for additional data acquisition for further analysis of the e-commerce food environment.

Food and beverage product information was captured using a website “scraping” [webscraping, which is an automated process used for extracting data from websites implemented using a bot or a web crawler (24)] program developed in Python. Each e-retailer's online website was first scanned to get a general outline of how the product information is stored on the website, followed by a Python-based routine to locate the hyperlinks of product pages. Once the hyperlinks are located, each product page was loaded, and its data was extracted into FLIP. The scraping was customized for each website. Developers analyzed the structure of the webpages, looking for common patterns to the way the data was displayed for each product. Random pages were selected for manual comparison to the results. Data that didn't make sense once imported into FLIP was compared to detailed logs captured during the scraping process. In all cases where the websites displayed data that was inconsistent with the data captured during scraping, it was confirmed by viewing the detailed logs. The data was then further processed with algorithms developed in C# programming language. A set of core classes and helper libraries provided the main functionality for data collection and processing while custom routines were developed to handle each e-retailer's unique website layout, page structures, webpage loading mechanisms, and data formats.

**Figure 2:**
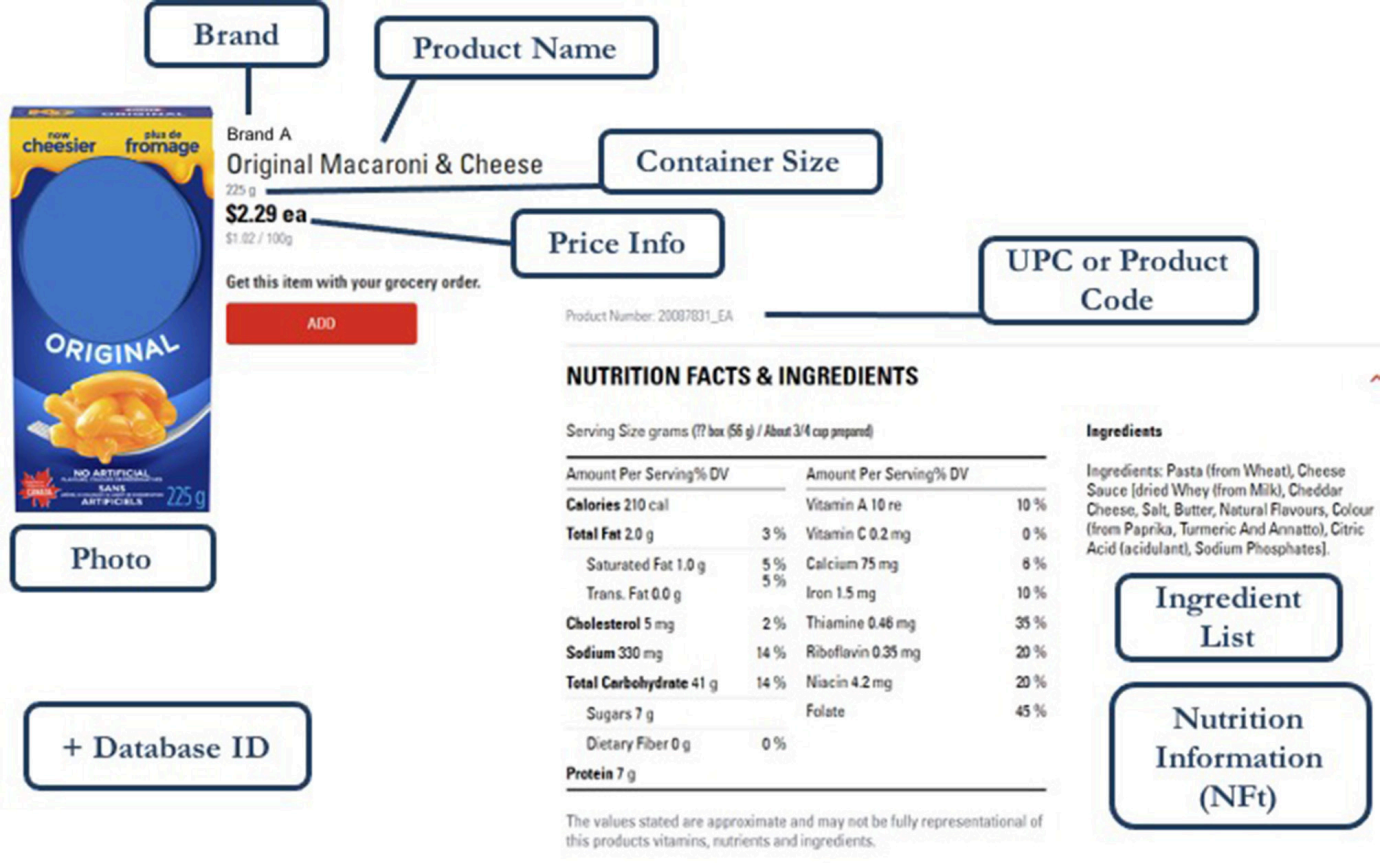
Example of the web-scraped information captured from the website of a major Canadian grocery retailer.

Every food product was collected, including all available national and private label brands, multiple sizes, and all flavors and varieties of a product. Information collected for each product included, where available, the following: product name, UPC, brand, NFt information, ingredients, container size, product image(s) as available, price (regular and sale price), dietary or allergen information (e.g., suitable for vegetarians) (if available on packaging as part of the ingredient list), and date and location/store information of sampling (Figure 2). Each product's UPC is used for identification of and tracking unique products over time.

### Phase 2: FLIP Data Capture and Management

After web-scraping the product information, foods were automatically assigned a product ID (an internal unique identifier) and photos and web data were uploaded onto the FLIP website for data management and processing. Each product's ID is used for identifying and tracking unique products over time. Artificial Intelligence (AI)-enhanced/powered Optical Character Recognition (OCR) (AI-enhanced OCR) technology was used to automatically extract data available in photo format only (e.g., NFt and ingredients list from certain websites). In the AI-enhanced OCR process, each image of the product was scanned for text and a text parsing algorithm determined which image had text that resembled the NFt or ingredient list, followed by extraction of that particular text only. All NFt data extracted from OCR technology (*n* = 7,400 products) were manually validated by FLIP staff and students, for this version to determine accuracy of the AI-OCR technology.

Barcodes of food products from Metro, Walmart, and Grocery Gateway and store-specific product numbers from Loblaws and No Frills in FLIP 2020 were matched to those in FLIP 2017 barcodes and store-specific product codes, respectively (*n* = 25,980). The no change in barcodes and product codes were used as indicators of no significant product change, therefore, the matching process allowed for any empty data fields in FLIP 2020 to be populated by FLIP 2017 (e.g., company name was not available on websites, but was determined in 2017 from package photos). However, food products from Costco, Walmart, Grocery Gateway, No Frills and Voilá could not be linked to FLIP 2017 as the previous versions of FLIP did not contain any Costco, Walmart, Grocery Gateway and No Frills products and Voilá did not contain any barcodes on the website. All product matches were manually validated by two Research Assistants and the following information was transferred over for the matched products: Table of Reference Amount (TRA) categories, sodium and sugar categories, Company/Parent Company, As Prepared NFts (nutrition facts information as per preparation required as specified on the product packaging but only for products if their NFts were identical), container size, serving size g/mL conversion factors (only if the package information for products was identical) and free sugars.

Barcodes of foods from FLIP 2020 were also linked to identical barcodes from FLIP 2020 from different stores (e.g., Kellogg's Cornflakes Family Size was linked across all webscraped stores given the barcode was identical). If one of these products was missing NFt or ingredients information, its data fields would be populated using a linked product with the most complete data. The FLIP log tracks when data is transferred from one product to another and the source.

### Phase 3: Data Processing and Food Categorization

In phase 3, food products were classified using Health Canada's Table of Reference Amounts (TRA) (32, 33), and two additional categories for therapeutic or supplemental products (e.g., meal replacements, nutritional supplements, vitamins) and variety packs (i.e., contain multiple products), followed by other multiple categorization systems (see Tables 1, 2). Health Canada's TRA categories consist of 24 major and 172 sub-categories, as well as an “other” category. Details on TRA categories can be found on Health Canada's website (33). TRA categorizations for unmatched products were applied using predictive algorithms, a method of AI-based estimation. All products with identical product names and brands were grouped together and given a predicted TRA category, powered by Artificial Intelligence/Machine Learning (AI/ML) predictive algorithms. Each product was then manually validated by FLIP staff and students.

Additional automation algorithms were developed for classifying foods into sodium-focused (34) and sugar-focused categories. Sodium-focused food categories were as follows: 13 food group categories, 52 major subcategories and 171 minor subcategories, as published in Health Canada's Guidance for the Food Industry on Reducing Sodium in Processed Foods (34). Sugar-focused categories were created, as described earlier (28, 35), consisting of 19 major food groups, 87 major subcategories, and 252 minor categories. All sugar and sodium categories were mapped manually to the TRA categories using the FLIP 2017 database as a guide. As a second step, keywords for each sodium and sugar category were manually created to assign products to particular categories (e.g., Toaster Strudel or Pop-Tarts as keywords for the category Toaster Pastries). Additional categories specific to various nutrient profiling models were also applied (e.g., FSANZ, Nutri-Score, PAHO etc.) (36–39).

For analyses requiring application of nutrient profiling models [models used to classify foods based on their nutrient composition (40)], foods and beverages in FLIP were categorized using the criteria established by the respective nutrient profiling model, verified independently by two research assistants, and any discrepancy resolved by consensus. The classification of FLIP products into each model's categories was based upon using a combination of information from TRA categories and subcategories (described above), sodium/sugar-focused categories, and the ingredient list. Products were also used to generate a list of foods and beverages with nutrition information and front-of-pack symbols (based on nutrient profiling model) for a FoodFlip^©^ smartphone application, as described in detail elsewhere (41). FoodFlip^©^ app categories consist of categorizing the FLIP database into product specific major categories (*n* = 19), sub-categories (*n* = 101) and minor categories (*n* = 397) in order to allow consumers to easily locate products in consumer-friendly categories. Categorization of foods for FoodFlip^©^ is based on merging Health Canada's TRA categories (33), Canada's sodium reformulation target categories [50], and more specific subsets of food categories [based on the iterative development process as described elsewhere (41)]. Categories were modified if found to be ambiguous or difficult for participants to understand or find during the reliability testing of the app.

For some products, serving sizes reported in milliliters were converted to grams for consistency across all products within a food category. Dependent upon specific research objectives and analyses, the database underwent quality control checks including verification of inputted nutrient contents using Atwater factors (i.e., checking for errors in nutrient declarations in the NFt, as determined by Atwater calculations where nutrients that were >20% from the declared caloric values were checked) and outliers to check for erroneous values.

**Figure 3:**
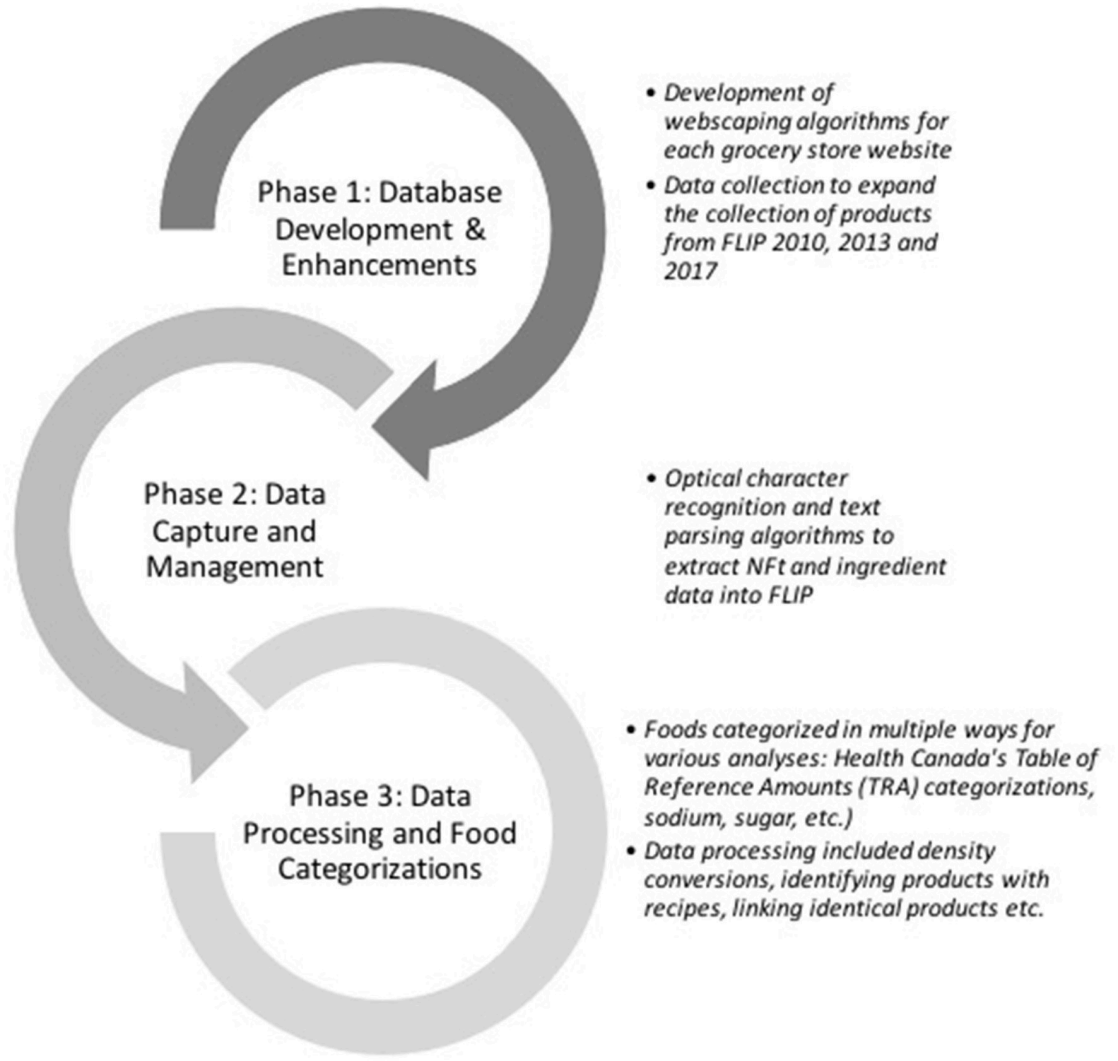
Database development, data management and processing of FLIP 2020. *Product information included product images, serving size, price, nutrition information, and ingredient list.^†^For this phase, for the purpose of method development and reliability. FLIP, Food Label Information Program (FLIP); NFt; Nutrition facts table.

Additional data extraction or processing, dependent on research objectives and analyses, are ongoing or will be conducted (e.g., application of nutrient profiling models, assessing marketing techniques, identification of nutrition claims and specific ingredients, calculation of free sugars content etc.) (Figure 3).

### FLIP Database in Other Countries

The development of the smartphone data collector app and web-based software has supported the establishment of FLIP databases in other countries including Argentina, Costa Rica, Paraguay and Peru, called FLIP for Latin American Countries or FLIP-LAC. Data from Argentina (*n* = 3,724) were collected between August 2017 and May 2018 from three leading groceries stores located in the province of Buenos Aires and Buenos Aires city (42). Costa Rican packaged food label data (*n* = 6,835) were collected from two grocery stores located in San Jose during and January-August 2018 (43), in addition to pilot data collected in the Summer of 2017. Data in Paraguay (*n* = 4,091) and Peruvian data (*n* = 1,533) were collected during the Summer 2017 and December 2019. The Canadian FLIP or the FLIP-LAC have been used for research, food supply monitoring, policy evaluation and modeling (28, 44–48).

## Results

A total of 74,445 products were collected from Metro, Costco, Walmart, Grocery Gateway (Longo's), Loblaws, Loblaw's Maple Leaf Gardens (a specific location in a metropolitan Toronto area), No Frills, No Frills Joe's (a specific location in a metropolitan Toronto area) and Voila. The number of products for each store was as follows: Metro (*n* = 11,268), Costco (*n* = 735), Walmart (*n* = 8,153), Grocery Gateway (*n* = 9,621), Loblaws (9,428), Loblaw's Maple Leaf Gardens (*n* = 9,414), No Frills (*n* = 6,603), No Frills Joe's (*n* = 6,764), and Voila (*n* = 12,459). However, food products from Loblaw's Maple Leaf Gardens (*n* = 9,414) and No Frills Joe's (*n* = 6,764) were omitted from the current analysis as they are duplicate outlets of the same data discussed in this manuscript. There were 1,261 (from seven retailers) and 1,409 (from nine retailers) non-food products (e.g., food intended solely for children under 4 years of age, meal replacements and nutritional supplements, alcohol), which were removed from further analysis. In total, 25,980 of the FLIP 2020 products (across all stores) were matched to 8,646 of the FLIP 2017 products. FLIP 2020 products may have been matched to multiple 2017 products, and vice versa. Therefore, the total number of matches was 26,395.

Of 73,036 food products, NFt were available for over 60% of products and data on ingredients were available for about 30% of the food and drinks. Data for energy, protein, carbohydrate, fat, sugars, sodium and saturated fat were present for about 65% of the products, data for fiber for 37%, while data for other nutrients were present for about 60% of the products (Table 3).

**Table 3:** Frequency and percentage of occurrence of data for nutrients collected in FLIP 2020.

**Nutrient**	**Number of products with nutrient data**	**% of products with specific nutrient data (*n* = 73,036 food downloads after removing non-foods)**	**% of products with specific nutrient data (*n* = 57,006 food downloads after No Frills and Loblaws duplicates removed)**
Energy	47,057	64.4%	58.9%
Total fat	44,479	60.9%	54.4%
Saturated fat	44,405	60.8%	54.2%
Protein	46,200	63.3%	57.4%
Total Carbohydrates	46,436	63.6%	57.8%
Total sugars	50,154	68.7%	64.3%
Fiber	27,239	37.3%	35.2%
Sodium	46,154	63.2%	57.3%
Ingredients	25,196	34.5%	44.2%

## Discussion

We developed a comprehensive product- and brand-specific database containing nutrition information for >70,000 foods and beverages sold by the largest Canadian food retailers, using web-scraping and OCR/AI capabilities. As consumers' eating patterns change toward an increased consumption of pre-packaged foods, branded food composition databases are a critical component for monitoring the packaged food supply, related to ongoing public health nutrition interventions and policy development (e.g., front of pack labeling, marketing to children, sodium reformulation, trans fat ban etc.).

Using automated techniques (e.g., webscraping, OCR with AI/ML) to collect data from e-grocery retailers can result in food composition databases with far greater coverage and temporality than have been achieved in the past (24), allowing for more detailed evaluation of the food supply system. Such large amounts of data require the development of automated procedures, but this level of granularity can also reveal insights about the constantly changing set of products available in the Canadian marketplace, including the rapid turnover and reformulation of products, and evaluating the real-time impact of food policies. The greater coverage allows for a comprehensive collection of the nutritional quality of foods available in the marketplace, and an assessment of the association between nutritional quality and other key variables that affect purchasing behavior, such as price. Analyses of this large and dynamic dataset can reveal insights such as the differences in fat, saturated fat, sugar and sodium between lower-priced and higher-priced ready products, and of the variability of available products, and changes in their composition over time. Such investigations have previously been conducted around the world and in Canada using past versions of the FLIP databases (i.e., FLIP 2010, 2013, and 2017) (26–28, 44, 46, 49–55).

The FLIP 2020 data collection via web-scraping showed that from about 73,000 foods, about 60% of products had NFt information, suggesting that automatically and repeatedly scraping data from online e-retailers websites can produce food composition databases with sufficient information on nutrients and ingredients with reliability to allow for monitoring/evaluating a highly dynamic food and beverage supply. In comparison, a study from UK on foodDB, with over 97,368 products, found data on specific nutrients were present for over 90% of nutrient declaration tables, with data on ingredients available for >80% of the foods and drinks (24). Considering that almost 30% of products had missing NFt/ingredient information in this study, this points to the need for policy or regulations on mandating retailers to provide food labeling information in the e-grocery retail environment in Canada to help consumers make healthy decisions when purchasing foods and beverages on these platforms.

The need for branded food databases as well as the challenges of creating such tools are recognized by researchers and policymakers (5, 6). These challenges include obtaining, updating and maintaining the database to accurately capture variation in product availability and formulation over time. Most importantly, once data collections and data input methods are automated, much more frequent collections become possible. New technology such as crowdsourcing, artificial intelligence and machine learning are the critical tools in addressing these challenges (24). These technologies enable a wider, more granular collection of food products, including capturing fresh and/or ready-to-eat foods. The AI/ML also has extended applications such as prediction of nutrients (e.g., added sugar, fiber) content in packaged foods using available nutrient, ingredient and food category information (56). Additionally, crowdsourced data allows for input of information on missing products and may provide a novel means for low-cost, real-time tracking of nutritional composition of the food supply, thereby enabling an expansion of the number of products captured (19). Notably, in many jurisdictions, e-retailers (e.g., online grocery/restaurants websites) are not required to provide nutrition information. Such discussions have begun at the CODEX Alimentarius Committee on Food Labeling (57). Given the impact of the COVID-19 pandemic on the uptake of online grocery shopping, which is likely to continue increasing in the upcoming years, it is essential for retailers to provide and for policymakers and researchers to be able to gather nutrition information at the (virtual) point of purchase. Therefore, collecting and maintaining current food nutrition information is a unique opportunity for health and nutrition researchers to collaborate with mathematicians and computer science specialists to develop faster and more reliable cloud-based databases. As an example, foodDB, using big data techniques, is a weekly updated database that collects data on a comprehensive sample of foods and beverages available for purchase in all major UK grocery stores (24). Another important evolution to gather data from the food supply is to request manufacturers provide digital food labels to centralized government databases. As an example, manufacturers in the United States already provide nutrition information to the USDA Food Branded Database, in text format (22). Adding copies or links to the digital food label from which nutrition information can be extracted using technology could enable ongoing data collection into the future.

## Limitations

Some limitations to our approach are related to the continued evolution and changes to the e-retailers product availability and websites, in order to ensure the data on these products are up-to-date. Although, the use web-scraping with OCR and AI/ML for data collected in FLIP 2020 were key innovations of our database that provided up-to-date product-specific nutrient information in a systematic and comprehensive manner, it was also a key limitation. E-grocery retailers may detect web-scraping, enabling OCR blockers and other techniques to make it difficult to scrape the data. Furthermore, there are no e-grocery food labeling regulations to mandate and standardize the availability and presentation of product information resulting in poor availability and wide inconsistencies in food labeling information, including missing information, number and quality of images, NFt and ingredients in the e-grocery retail environment in Canada (58).

The current FLIP2020 does not capture local regional and geographical variability of food and drink availability within individual online grocery retailers nor does it capture regional and local ethnic supermarkets that once catered to immigrant communities but are serving non-immigrant consumers seeking new products. Furthermore, convenience stores and large drugstore chains are introducing new product ranges that often include foods, which are not currently captured by the FLIP database.

Some tasks needed for research or monitoring remain time- and labor-intensive. For example, creating scores for some nutrient profiling models, automatic mapping of categories and subcategories and parsing of ingredients in any database remain, although work is underway to apply AI/ML to such tasks.

## Strengths

The automation of FLIP 2020 is a first step in providing real time nutritional data on foods. Web-scraping coupled with AI-powered OCR technology are important tools in automating the collection of real-time foods and nutrition information. The automated data collect process, using AI-enhanced OCR, provides FLIP with distinct analytical advantages compared to previous versions of FLIP and the generic food composition database in Canada (CNF) and takes the burden off manual processing by staff and students. A systematic methodology was established, based on previous versions of FLIP, to validate and categorize information, thereby enhancing the collection, storage, processing and management of nutrition information for each product. The use of web-scraping and automation further lowers the cost for future collections and allows for regularity in data capture on products. These features can also be implemented for future collections of FLIP databases, such as the FLIP-LAC and can be useful for other nutrition databases. Automating the systematic and consistent data capture will ensure sustainability and feasibility of maintaining large-scale branded food composition databases as new products and other changes to product formulation are introduced and others discontinued.

## Conclusion

FLIP 2020 is an automated methodological step forward for food composition databases, which are the bedrock of nutritional epidemiology. Web-scraping coupled with OCR technology (AI/ML) are important tools in automating the collection of real-time food and nutrition information. The FLIP 2020 data collection demonstrated that automatically scraping data from online supermarkets can produce a food composition database with sufficient accuracy, transparency, granularity and flexibility to regularly monitor a highly dynamic food and drink marketplace. Such information are important in understanding the relationships between the nutritional quality of food products and measurements of policy impacts and health over time.

## Data Availability Statement

The datasets presented in this article are not readily available because complete database for non-commercial use can be obtained from the corresponding author at mary.labbe@utoronto.ca through data sharing agreements. Requests to access the datasets should be directed to Mary L'Abbe, mary.labbe@utoronto.ca.

## Author Contributions

MA, AS, and ML'A conceived and designed the study. AS, JL, and MW collected the data. MA, AS, JL, MW, and BF-A processed the different phases of the FLIP database. All authors contributed to interpreting the data as well as writing and editing the manuscript and have read and agreed to the published version of the manuscript.

## Funding

This research was supported by funds from the Canadian Institute of Health Research Project Grant - Policy Impact (2016PJT-378415)(ML'A). MA was supported by a Postdoctoral Fellowship from the Joannah and Brian Lawson Center for Child Nutrition at University of Toronto, Toronto, ON, Canada. JL was supported by CIHR Doctoral Fellowship and the Banting and Best Diabetes Center at University of Toronto, Toronto, ON, Canada. BF-A was supported by a Postdoctoral Fellowship at Ontario Tech University. MW was supported by a Banting and Best Graduate Studentship Award.

## Conflict of Interest

ML'A and MA report receiving a competitive research grant from IAFNS formerly ILSI NA to analyze NHANES data to determine the intakes and sources of sodium in the diets of Americans 2021–2022. None of these companies/organizations had any involvement in the present research. The remaining authors declare that the research was conducted in the absence of any commercial or financial relationships that could be construed as a potential conflict of interest.

## Publisher's Note

All claims expressed in this article are solely those of the authors and do not necessarily represent those of their affiliated organizations, or those of the publisher, the editors and the reviewers. Any product that may be evaluated in this article, or claim that may be made by its manufacturer, is not guaranteed or endorsed by the publisher.
